# Incidence of Type 1 Diabetes Mellitus in Turkish Children from the Southeastern Region of the Country: A Regional Report

**DOI:** 10.4274/Jcrpe.954

**Published:** 2013-05-30

**Authors:** Hüseyin Demirbilek, Mehmet Nuri Özbek, Rıza Taner Baran

**Affiliations:** 1 Diyarbakır Children State Hospital, Division of Pediatric Endocrinology, Diyarbakır, Turkey

**Keywords:** type 1 diabetes mellitus, incidence, diabetic ketoacidosis, Epidemiology

## Abstract

**Objective:** Variability in the incidence of type 1 diabetes mellitus (T1DM) related to geographical region, ethnic background, gender, and age indicates a need for further epidemiological studies. To date, there are no reported studies on the incidence of T1DM in the pediatric age group from the Southeastern region of Turkey. To define the incidence, demographic and clinical characteristics of T1DM in children 0-14 years of age in Diyarbakir, one of the largest cities in the Southeast region of Turkey.

**Methods:** Hospital files of patients with the diagnosis of T1DM were reviewed. Data of all patients diagnosed between 1 June 2010 and 31 May 2011 were evaluated. Population data on the 0-14 age group were obtained from the Turkish Statistical Institute (TSI) reports.

**Results:** From a total of 41 T1DM patients, 24 (58.5%) were female (male: 41.5%) with a male/female ratio of 1.4. The overall annual incidence of T1DM was 7.2/105, being 8.7/105 in females and 5.7/105 in males. The peak incidence was found to occur at age 5-9 years in the girls and 10-14 years in the boys. Mean age at diagnosis was 8.1±3.8 years. Rate of presentation with diabetic ketoacidosis was 65.9%. Patients applied most frequently in spring and winter months.

**Conclusions:** In this first T1DM incidence study on the pediatric age group in Diyarbakir, Turkey, T1DM incidence was found to be similar to that in countries with low-middle incidence.

**Conflict of interest:**None declared.

## INTRODUCTION

Type 1 diabetes mellitus (T1DM) is the most frequently encountered chronic disease of childhood. It is known that T1DM incidence is increasing among children all around the world. However, reported figures for T1DM incidence in childhood indicate a large variability among different populations (1,2,3,4,5,6,7,8,9,10,11). This variability is explained by differences in ethnic background, geographical region, and level of industrial development (9,10,11,12,13,14,15,16,17). Turkey is a country midway between Europe and Asia and contains a variety of communities within its geographic borders. A nationwide registry for pediatric T1DM patients has been instituted only recently, and data on incidence of T1DM are very limited. The 5th edition of the world diabetes atlas, published in 2011 by the International Diabetes Federation (IDF), contains no data on incidence of pediatric diabetes in Turkey (18).

To the best of our knowledge, there are no published studies on incidence of T1DM in children living in the Southeastern region of Turkey. In this present study, we aimed to report on the annual incidence of T1DM in children below 14 years of age in Diyarbakir, one of the largest cities in this region, and also to describe the demographic and clinical characteristics of these patients. 

## METHODS

Diyarbakir Children’s Hospital is the only pediatric endocrine clinic in the city of Diyarbakir which is authorized to write report for insulin for children with T1DM that can be funded by the Social Security Institution (SSI). Therefore, all patients with a diagnosis of T1DM in Diyarbakir who are under 14 years of age and whose parents are beneficiaries of SSI (a large fraction of the population) are referred to our clinic.

Medical records of all T1DM patients diagnosed between 1 June 2010 and 31 May 2011 were reviewed. The number of newly diagnosed patients was calculated, from these records. The distribution of patients in the different districts of Diyarbakir city was also determined.

Diagnosis of T1DM was made according to the criteria of the American Diabetes Association (ADA). Blood glucose level, pH, HCO3 level, glycosylated hemoglobin (HbA1c) value, and presence of ketosis or ketoacidosis at the time of diagnosis were analyzed. Insulin and C-peptide levels were measured in all patients before initiation of insulin treatment. The records also provided information on clinical signs at presentation (acute onset, weight loss, ketosis/ketoacidosis, family history, etc.). Patients who had a venous blood pH<7.30 or bicarbonate <15 mEq/L were accepted to have diabetic ketoacidosis (DKA). Low levels of insulin and C-peptide accompanying a high blood glucose level at the time of diagnosis were accepted as supportive findings for T1DM diagnosis.

## STATISTICAL ANALYSIS

SPSS 11.0 for Windows® software package program was used in the analysis. Data were expressed as mean±SD (range) or median (25-75 interquartile range). Mann-Whitney U test was used in comparing medians, whereas Chi-square test was used in comparing the ratios. A p-value of less than 0.05 was accepted as statistically significant.

## RESULTS

A diagnosis of T1DM was made in a total of 41 patients in the 0-14-year age group in the course of one year time starting on 1 June 2010 and ending on 31 May 2011. According to the home address based census records of the Turkish Statistical Institute (TSI) for year 2010, the total population in Diyarbakir was 1528958 and the total population in the 0-14-year age group was 565386. Calculated T1DM incidence based on these data was 7.2/105/year. Distribution of population according to gender and age groups, numbers of female and male patients with diabetes, and calculated incidence based on these data are shown in Table 1. The peak incidence in girls was noted at age 5-9 years, while the highest incidence in boys was between ages 10 and 14 years (Table 1). While the incidence in girls and boys were similar in the 0-4-year age group, the incidence in girls was higher in all older age groups, the difference being most striking in age group 5-9 years.

**Figure 1:**
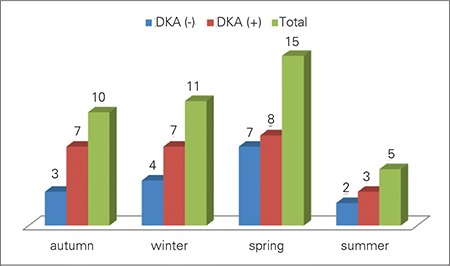
Distribution of patients according to presentation by seasonof the year and frequency of diabetic ketoacidosis (DKA)

Of a total of 41 patients, 26 were from central Diyarbakir, 7 were from its districts, and 8 were from villages in the vicinity of the city. Since we had no access to detailed data on number of children of 0-14-year age group living in the city centrum, districts and villages, we could not calculate the incidence rates separately. Total numbers of female and male patients were 24 (58.5%) and 17 (41.5%), respectively, and the female/male ratio was 1.4 in favor of girls. Parents of 23 patients (56.1%) were first-degree cousins. In the family history of diabetes, 11 patients (26.8%) had T1DM and 16 patients (39%) had T2DM in their parents or relatives, so that a total of 25 patients (61%) had T1DM or T2DM in their family history. When patients were examined according to the season of the year at diagnosis, the most frequent time of presentation was in the spring (Figure 1). All patients in the series had social security coverage. Mean (range) number of siblings of the patient group was 5.

Mean age at diagnosis was 8.1±3.8 years (range: 1.67-14.0). Clinical and laboratory characteristics of the patients at admission are given in Table 2.

Of 41 patients, 27 (65.9%) presented with DKA. In this group, 2 patients (7.4%) had mild (pH: 7.3-7.2), 8 patients (29.6%) had moderate (pH:7.2-7.1), and 17 (63%) had severe (pH<7.1) acidosis. When the frequency of DKA was evaluated by age groups, 7 out of 8 patients (87.5%) in the 0-4-year age group were found to present with DKA. This frequency was 9 out of 17 patients (52.9%) in the 5-9-year age group and 11 out of 16 patients (68.7%) in the 10-14-year age group (p=0.042). Twenty seven patients (65.9%) were prepubertal at diagnosis and 14 (34.1%) were pubertal. There was no statistically significant difference between the prepubertal and pubertal children regarding frequency of DKA at presentation (66.7% and 64.3%, respectively; p=0.879). There was no statistically significant difference in presenting with DKA between patients according to season of the year (p=0.981). Frequency of presentation with DKA was lower in patients with a positive family history of DM (60%) than in those with a negative family history of DM (75%), but the difference was not significant (p=0.160). Although, presentation with DKA was more frequent in the villages as compared to the districts and centrum of Diyarbakir city, the difference was not statistically significant (presentation with DKA was 75%; 71.4% and 61.5%, respectively; p=0.737). There was no statistically significant difference between median age, blood glucose level, insulin levels, C-peptide levels, the number of siblings, and HbA1c levels of patients presenting with and without DKA (Table 3). 

## DISCUSSION

In this study, T1DM incidence in the 0-14-year age group was determined as 7.2/105/year in Diyarbakir, which is one of the largest cities in the Southeast region of Turkey. This study, which is the first on local incidence performed on the pediatric age group in Turkey, demonstrates that T1DM incidence in children of Diyarbakir is similar to that in countries in the “low-middle incidence” group.

The highest incidence figures for T1DM in children have been reported from Scandinavian countries such as Finland, Scotland and Sweden (4,10,16). With the exception of the Sardinia region in Italy, European countries tend to have lower incidence rates than the Scandinavian countries (United Kingdom 29.8/105; Czech Republic 17.2/105; Germany 18.3/105). Even lower rates have been reported from Slovenia (11.1/105), Slovakia (13.6/105), and eastern European countries like Greece (9.9/105), Croatia (8.9/105), Bulgaria (9.4/105), Albania (3.9/105), and Bosnia Herzegovina (3.5/105) (4,7). As expected, T1DM incidence detected in Diyarbakir was lower than in Finland and other Scandinavian countries as well being lower than in many European countries and was similar to the incidence rates reported from neighboring countries in Eastern Europe (4,6,7,19,20,21).

T1DM incidence in Diyarbakir was also low as compared to figures reported from the USA, Australia, Canada, and New Zealand and similar to those reported from South American countries like Chile (7-8/105) and Brazil (6.3-10/105) (4,10,22,23). As expected, the incidence rate in our pediatric population was higher than that reported from China, Japan, Iran, and Venezuela, but somehow was lower than the rates reported from Saudi Arabia (27.5/105) and Kuwait (20.1/105) (4,10,18,24,25). The incidence reported from Russia for children in Moscow was higher (12.9/105) than that in our sample (26). The largest T1DM prevalence study from Turkey in the pediatric age group was conducted by Akesen et al (27) in 2009 and comprised 1630751 school-aged children (range: 6-18 years) from Istanbul. These authors reported a T1DM prevalence of 0.67/1000, which was lower than the USA, North Europe, Kuwait, and Saudi Arabia figures but similar to the estimates reported for Balkan and Mediterranean countries excluding Sardinia in Italy.

Although we did not have the means to calculate T1DM incidence rates of children living in the center of Diyarbakir city, its districts, and in nearby villages separately, we found a great difference in the incidence between urban children (city center plus districts) and those coming from rural (villages) areas. These findings were in line with previous literature data indicating that urbanized life style caused an increased T1DM rate when compared to the rural life style. This difference has been attributed to having more chance of encountering environmental factors promoting development of T1DM in an urbanized environment (28,29,30,31,32). In an ecological analysis on the incidence of childhood T1DM in Europe, it was reported that the incidence was positively correlated with national indicators of prosperity, such as gross domestic product and low infant mortality (33). Since the exact mechanism of how urbanization and wealth affect the frequency of T1DM remains unclear, further evaluation are needed.

T1DM incidence differs also by age groups. It is known that the incidence shows a peak generally in the peripubertal period (10-14-year age group). However, currently there are several studies from Turkey and from other countries which report T1DM diagnosis in patients of a younger age (4,21,34,35,36). The peak incidence of the disease is reported to occur in the 5-9-year age group in some countries, especially those where T1DM is more frequently encountered, such as Finland and Sweden (4,25,36). Excluding African countries, the disease peaks generally earlier in girls than boys (37). In our study also, age of peak incidence was 5-9 years, but this finding was due to the higher numbers of girl patients and the earlier age at onset of T1DM in girls. Peak incidence age was reported as 10-14 years in studies from Turkey, but small peaks at earlier ages have also been reported in these studies (38,39).

Although female/male ratio in T1DM patients is generally reported to be equal, there are a number of studies which report that the condition might be slightly frequent either in boys or in girls (10). Overall, the frequency is reported to be higher in boys from high incidence countries and higher in girls from low incidence countries (40,41,42). Our results appear to be in line with the literature data.

As to the clinical characteristics of our patients at presentation, DKA was found to be at a high rate of 65%. This ratio was higher than the DKA rates reported in cross-sectional studies (33% and 43.1%)from Turkey (38,39,43)and from European countries (12,26,44,45). It has also been reported that the rate of presentation with DKA tends to decrease over time due to increased awareness on the part of the parents (45,46,47). Nearly all of our patients in the 0-4-year age group (87.5%) presented with DKA, and presentation with DKA was higher in our study compared to other reports from Turkey. Having no social security coverage was stated as one reason for high rate of presentation with DKA in Turkey. This was not true for our patients since all had social security coverage. The high rate of DKA at presentation might have been due to the low socioeconomic structure of the region as a whole. In line with this reasoning, the DKA rate was higher in patients from villages compared to those from the city center, although the difference was not statistically significant. Rate of presentation with DKA was lower in patients with a family history of DM, supporting the notion that awareness of diabetes might have an effect in decreasing presentation with DKA. Pubertal status or season had no effect on DKA rates.

In conclusion, T1DM incidence among children in the 0-14-year age group was determined as 7.2/105 in Diyarbakir, which is one of the biggest cities in the Southeast region of Turkey. Further local incidence studies as well as large scale studies covering the whole country should be conducted to define the nationwide T1DM incidence and related health data in Turkey. 

## Figures and Tables

**Table 1:**
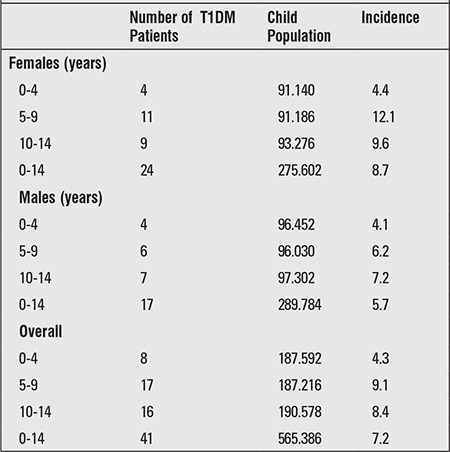
Calculated incidence of type 1 diabetes mellitus (T1DM) infemale and male children of different age groups within the 0-14-yearage group in Diyarbakir city

**Table 2:**
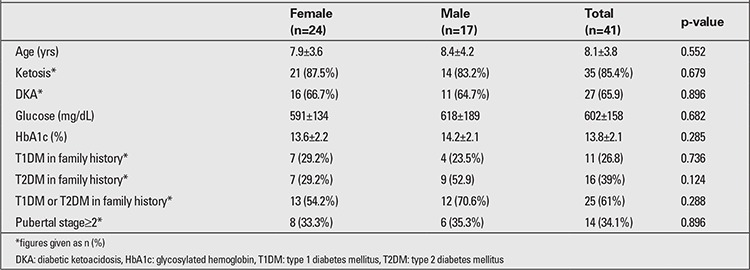
Clinical and laboratory characteristics of female and male patients at the time of admission

**Table 3:**
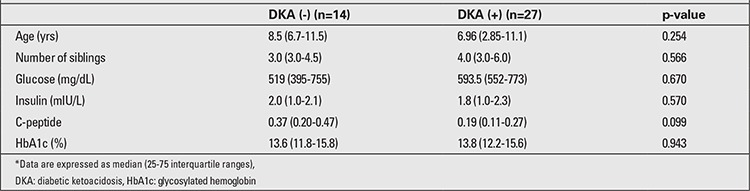
Clinical and laboratory characteristics of patients who presented with or without diabetic ketoacidosis*
